# 5-Bromo-3-(4-fluoro­phenyl­sulfin­yl)-2,7-dimethyl-1-benzofuran

**DOI:** 10.1107/S1600536810049986

**Published:** 2011-01-12

**Authors:** Hong Dae Choi, Pil Ja Seo, Byeng Wha Son, Uk Lee

**Affiliations:** a Department of Chemistry, Dongeui University, San 24 Kaya-dong Busanjin-gu, Busan 614-714, Republic of Korea; b Department of Chemistry, Pukyong National University, 599-1 Daeyeon 3-dong, Nam-gu, Busan 608-737, Republic of Korea

## Abstract

In the title compound, C_16_H_12_BrFO_2_S, the 4-fluoro­phenyl ring makes a dihedral angle of 83.29 (5)° with the mean plane of the benzofuran fragment. In the crystal, mol­ecules are linked by weak inter­molecular C—H⋯O hydrogen bonds.

## Related literature

For the pharmacological activity of benzofuran compounds, see: Aslam *et al.* (2006 ▶); Galal *et al.* (2009 ▶); Khan *et al.* (2005 ▶). For natural products with benzofuran rings, see: Akgul & Anil (2003 ▶); Soekamto *et al.* (2003 ▶). For our previous structural studies of related 3-[(4-fluoro­phen­yl)sulfin­yl]-5-halo-2-methyl-1-benzofuran derivatives, see: Choi *et al.* (2010*a* ▶,*b* ▶,*c* ▶).
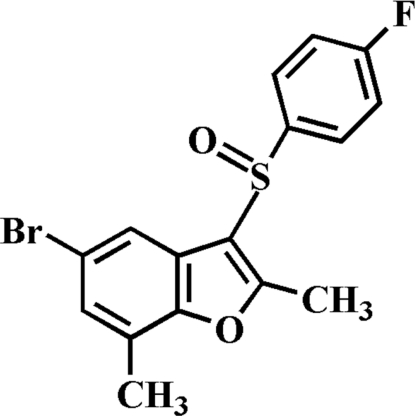


## Experimental

### 

#### Crystal data

C_16_H_12_BrFO_2_S
*M* *_r_* = 367.23
Triclinic, 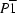

*a* = 7.6696 (1) Å
*b* = 8.6308 (1) Å
*c* = 12.3225 (2) Å
α = 96.084 (1)°
β = 91.510 (1)°
γ = 113.609 (1)°
*V* = 741.08 (2) Å^3^
*Z* = 2
Mo *K*α radiation
μ = 2.93 mm^−1^
*T* = 173 K
0.26 × 0.16 × 0.13 mm

#### Data collection

Bruker SMART APEXII CCD diffractometer
Absorption correction: multi-scan (*SADABS*; Bruker, 2009 ▶) *T*_min_ = 0.583, *T*_max_ = 0.746
13338 measured reflections
3422 independent reflections
2976 reflections with *I* > 2σ(*I*)
*R*_int_ = 0.029

#### Refinement

*R*[*F* ^2^ > 2σ(*F* ^2^)] = 0.028
*wR*(*F* ^2^) = 0.074
*S* = 1.07
3422 reflections
192 parameters
H-atom parameters constrained
Δρ_max_ = 0.33 e Å^−3^
Δρ_min_ = −0.56 e Å^−3^

### 

Data collection: *APEX2* (Bruker, 2009 ▶); cell refinement: *SAINT* (Bruker, 2009 ▶); data reduction: *SAINT*; program(s) used to solve structure: *SHELXS97* (Sheldrick, 2008 ▶); program(s) used to refine structure: *SHELXL97* (Sheldrick, 2008 ▶); molecular graphics: *ORTEP-3* (Farrugia, 1997 ▶) and *DIAMOND* (Brandenburg, 1998 ▶); software used to prepare material for publication: *SHELXL97*.

## Supplementary Material

Crystal structure: contains datablocks global, I. DOI: 10.1107/S1600536810049986/zq2078sup1.cif

Structure factors: contains datablocks I. DOI: 10.1107/S1600536810049986/zq2078Isup2.hkl

Additional supplementary materials:  crystallographic information; 3D view; checkCIF report

## Figures and Tables

**Table 1:** Hydrogen-bond geometry (Å, °)

*D*—H⋯*A*	*D*—H	H⋯*A*	*D*⋯*A*	*D*—H⋯*A*
C13—H13⋯O2^i^	0.95	2.41	3.259 (2)	148

Symmetry code: (i) 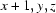.

**Fig. 1.**
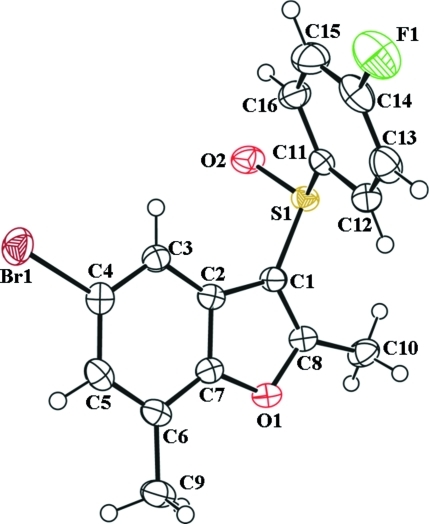
The molecular structure of the title compound with the atom numbering scheme. Displacement ellipsoids are drawn at the 50% probability level. H atoms are presented as small spheres of arbitrary radius.

## supplementary crystallographic information

### Comment

A series of benzofuran ring system exhibit interesting potent pharmacological properties such as antifungal, antitumor and antiviral, antimicrobial activities (Aslam *et al.*, 2006; Galal *et al.*, 2009; Khan *et al.*, 2005). These compounds widely occur in nature (Akgul & Anil, 2003; Soekamto *et al.*, 2003). As a part of our study of the substituent effect on the solid state structures of 3-[(4-fluorophenyl)sulfinyl]-5-halo-2-methyl-1-benzofuran analogues (Choi *et al.*, 2010**a*,*b*,c*), we report here the crystal structure of the title compound

In the title molecule, the benzofuran unit is essentially planar, with a mean deviation of 0.011 (1) Å from the least-squares plane defined by the nine constituent atoms (Fig. 1). The dihedral angle formed by the mean plane of the benzofuran fragment and the 4-fluorophenyl ring is 83.29 (5)°. The molecular packing is stabilized by weak intermolecular C—H···O hydrogen bonds between the 4-fluorophenyl H13 atom and the oxygen of the S═O unit (Table 1; C13—H13···O2^i^).

### Experimental

77% 3-chloroperoxybenzoic acid (202 mg, 0.9 mmol) was added in small portions to a stirred solution of 5-bromo-3-[(4-fluorophenyl)sulfanyl]-2,7-dimethyl-1-benzofuran (281 mg, 0.8 mmol) in dichloromethane (40 mL) at 273 K. After being stirred at room temperature for 3h, the mixture was washed with saturated sodium bicarbonate solution and the organic layer was separated, dried over magnesium sulfate, filtered and concentrated at reduced pressure. The residue was purified by column chromatography (hexane–ethyl acetate, 1:1 v/v) to afford the title compound as a colorless solid [yield 75%, m.p. 407–408 K; *R*_f_ = 0.67 (hexane-ethyl acetate, 1:1 v/v)]. Single crystals suitable for X-ray diffraction were prepared by slow evaporation of a solution of the title compound in acetone at room temperature.

### Refinement

All H atoms were positioned geometrically and refined using a riding model, with C—H = 0.95 Å for aryl and 0.98 Å for methyl H atoms. *U*_iso_(H) = 1.2*U*_eq_(C) for aryl and 1.5*U*_eq_(C) for methyl H atoms.

### Crystal data

**Table:**

C_16_H_12_BrFO_2_S	*Z* = 2
*M**_r_* = 367.23	*F*(000) = 368
Triclinic, *P*1	*D*_x_ = 1.646 Mg m^−^^3^
Hall symbol: -P 1	Mo *K*α radiation, λ = 0.71073 Å
*a* = 7.6696 (1) Å	Cell parameters from 6925 reflections
*b* = 8.6308 (1) Å	θ = 2.6–27.4°
*c* = 12.3225 (2) Å	µ = 2.93 mm^−^^1^
α = 96.084 (1)°	*T* = 173 K
β = 91.510 (1)°	Block, colourless
γ = 113.609 (1)°	0.26 × 0.16 × 0.13 mm
*V* = 741.08 (2) Å^3^	

### Data collection

**Table:**

Bruker SMART APEXII CCD diffractometer	3422 independent reflections
Radiation source: rotating anode	2976 reflections with *I* > 2σ(*I*)
graphite multilayer	*R*_int_ = 0.029
Detector resolution: 10.0 pixels mm^-1^	θ_max_ = 27.6°, θ_min_ = 1.7°
φ and ω scans	*h* = −8→9
Absorption correction: multi-scan (*SADABS*; Bruker, 2009)	*k* = −11→11
*T*_min_ = 0.583, *T*_max_ = 0.746	*l* = −16→16
13338 measured reflections	

### Refinement

**Table:**

Refinement on *F*^2^	Primary atom site location: structure-invariant direct methods
Least-squares matrix: full	Secondary atom site location: difference Fourier map
*R*[*F*^2^ > 2σ(*F*^2^)] = 0.028	Hydrogen site location: difference Fourier map
*wR*(*F*^2^) = 0.074	H-atom parameters constrained
*S* = 1.07	*w* = 1/[σ^2^(*F*_o_^2^) + (0.0397*P*)^2^ + 0.2033*P*] where *P* = (*F*_o_^2^ + 2*F*_c_^2^)/3
3422 reflections	(Δ/σ)_max_ < 0.001
192 parameters	Δρ_max_ = 0.33 e Å^−^^3^
0 restraints	Δρ_min_ = −0.56 e Å^−^^3^

### Special details

**Table:**

Geometry. All esds (except the esd in the dihedral angle between two l.s. planes) are estimated using the full covariance matrix. The cell esds are taken into account individually in the estimation of esds in distances, angles and torsion angles; correlations between esds in cell parameters are only used when they are defined by crystal symmetry. An approximate (isotropic) treatment of cell esds is used for estimating esds involving l.s. planes.
Refinement. Refinement of F^2^ against ALL reflections. The weighted R-factor wR and goodness of fit S are based on F^2^, conventional R-factors R are based on F, with F set to zero for negative F^2^. The threshold expression of F^2^ > 2sigma(F^2^) is used only for calculating R-factors(gt) etc. and is not relevant to the choice of reflections for refinement. R-factors based on F^2^ are statistically about twice as large as those based on F, and R- factors based on ALL data will be even larger.

### Fractional atomic coordinates and isotropic or equivalent isotropic displacement parameters (Å^2^)

**Table:**

	*x*	*y*	*z*	*U*_iso_*/*U*_eq_	
Br1	−0.07124 (3)	0.06945 (2)	0.199937 (19)	0.04181 (9)	
S1	0.18515 (6)	0.85094 (6)	0.34130 (4)	0.02781 (11)	
F1	0.6274 (2)	0.6390 (2)	0.64210 (12)	0.0602 (4)	
O1	0.32437 (18)	0.74870 (16)	0.04617 (10)	0.0286 (3)	
O2	−0.01904 (19)	0.75643 (19)	0.36136 (12)	0.0377 (3)	
C1	0.2264 (3)	0.7600 (2)	0.21574 (14)	0.0258 (4)	
C2	0.1739 (2)	0.5831 (2)	0.17564 (14)	0.0246 (4)	
C3	0.0805 (2)	0.4290 (2)	0.21663 (15)	0.0264 (4)	
H3	0.0370	0.4241	0.2882	0.032*	
C4	0.0547 (3)	0.2838 (2)	0.14748 (16)	0.0290 (4)	
C5	0.1195 (3)	0.2869 (3)	0.04259 (16)	0.0306 (4)	
H5	0.0975	0.1823	−0.0011	0.037*	
C6	0.2153 (2)	0.4392 (3)	0.00081 (15)	0.0282 (4)	
C7	0.2371 (2)	0.5836 (2)	0.07052 (14)	0.0247 (4)	
C8	0.3136 (3)	0.8532 (2)	0.13575 (15)	0.0278 (4)	
C9	0.2924 (3)	0.4477 (3)	−0.11043 (16)	0.0371 (5)	
H9A	0.2846	0.5445	−0.1420	0.056*	
H9B	0.2169	0.3418	−0.1582	0.056*	
H9C	0.4258	0.4622	−0.1036	0.056*	
C10	0.3976 (3)	1.0383 (3)	0.12828 (18)	0.0372 (5)	
H10A	0.3640	1.0983	0.1908	0.056*	
H10B	0.3477	1.0599	0.0601	0.056*	
H10C	0.5367	1.0793	0.1290	0.056*	
C11	0.3197 (3)	0.7776 (2)	0.42827 (14)	0.0266 (4)	
C12	0.5103 (3)	0.8142 (3)	0.41325 (17)	0.0339 (4)	
H12	0.5684	0.8718	0.3536	0.041*	
C13	0.6149 (3)	0.7668 (3)	0.48527 (18)	0.0392 (5)	
H13	0.7453	0.7904	0.4762	0.047*	
C14	0.5250 (3)	0.6845 (3)	0.57035 (17)	0.0392 (5)	
C15	0.3374 (3)	0.6460 (3)	0.58663 (18)	0.0432 (5)	
H15	0.2798	0.5873	0.6459	0.052*	
C16	0.2333 (3)	0.6950 (3)	0.51448 (16)	0.0348 (4)	
H16	0.1031	0.6717	0.5244	0.042*	

### Atomic displacement parameters (Å^2^)

**Table:**

	*U*^11^	*U*^22^	*U*^33^	*U*^12^	*U*^13^	*U*^23^
Br1	0.04528 (14)	0.02586 (12)	0.05240 (15)	0.01188 (9)	0.00989 (10)	0.00587 (9)
S1	0.0333 (2)	0.0265 (2)	0.0255 (2)	0.01415 (19)	0.00559 (18)	0.00215 (18)
F1	0.0650 (9)	0.0718 (10)	0.0519 (8)	0.0382 (8)	−0.0190 (7)	0.0048 (7)
O1	0.0308 (6)	0.0331 (7)	0.0241 (6)	0.0142 (5)	0.0064 (5)	0.0068 (5)
O2	0.0312 (7)	0.0510 (9)	0.0346 (7)	0.0200 (6)	0.0082 (6)	0.0056 (6)
C1	0.0290 (9)	0.0263 (9)	0.0236 (8)	0.0124 (7)	0.0040 (7)	0.0042 (7)
C2	0.0240 (8)	0.0293 (9)	0.0230 (8)	0.0135 (7)	0.0009 (7)	0.0031 (7)
C3	0.0270 (8)	0.0279 (9)	0.0255 (9)	0.0120 (7)	0.0018 (7)	0.0053 (7)
C4	0.0267 (9)	0.0270 (9)	0.0346 (10)	0.0124 (7)	−0.0004 (7)	0.0035 (8)
C5	0.0271 (9)	0.0336 (10)	0.0318 (10)	0.0157 (8)	−0.0040 (7)	−0.0047 (8)
C6	0.0239 (8)	0.0395 (11)	0.0235 (8)	0.0167 (8)	−0.0019 (7)	−0.0011 (8)
C7	0.0227 (8)	0.0302 (9)	0.0235 (8)	0.0126 (7)	0.0009 (7)	0.0064 (7)
C8	0.0290 (9)	0.0306 (10)	0.0261 (9)	0.0140 (7)	0.0046 (7)	0.0056 (7)
C9	0.0351 (10)	0.0512 (13)	0.0255 (9)	0.0193 (9)	0.0026 (8)	−0.0012 (9)
C10	0.0433 (11)	0.0316 (11)	0.0391 (11)	0.0152 (9)	0.0136 (9)	0.0122 (9)
C11	0.0300 (9)	0.0232 (9)	0.0234 (8)	0.0087 (7)	0.0027 (7)	−0.0016 (7)
C12	0.0316 (9)	0.0348 (11)	0.0311 (10)	0.0097 (8)	0.0065 (8)	0.0003 (8)
C13	0.0309 (10)	0.0441 (12)	0.0408 (12)	0.0166 (9)	−0.0021 (9)	−0.0068 (9)
C14	0.0463 (12)	0.0398 (12)	0.0335 (11)	0.0227 (10)	−0.0119 (9)	−0.0044 (9)
C15	0.0478 (12)	0.0501 (13)	0.0329 (11)	0.0195 (10)	0.0034 (9)	0.0121 (10)
C16	0.0315 (10)	0.0411 (11)	0.0306 (10)	0.0123 (8)	0.0061 (8)	0.0085 (8)

### Geometric parameters (Å, °)

**Table:**

Br1—C4	1.9047 (19)	C8—C10	1.479 (3)
S1—O2	1.4896 (14)	C9—H9A	0.9800
S1—C1	1.7542 (18)	C9—H9B	0.9800
S1—C11	1.796 (2)	C9—H9C	0.9800
F1—C14	1.355 (2)	C10—H10A	0.9800
O1—C8	1.376 (2)	C10—H10B	0.9800
O1—C7	1.381 (2)	C10—H10C	0.9800
C1—C8	1.355 (3)	C11—C16	1.377 (3)
C1—C2	1.440 (2)	C11—C12	1.388 (3)
C2—C3	1.391 (2)	C12—C13	1.379 (3)
C2—C7	1.395 (2)	C12—H12	0.9500
C3—C4	1.379 (3)	C13—C14	1.372 (3)
C3—H3	0.9500	C13—H13	0.9500
C4—C5	1.396 (3)	C14—C15	1.367 (3)
C5—C6	1.387 (3)	C15—C16	1.385 (3)
C5—H5	0.9500	C15—H15	0.9500
C6—C7	1.385 (3)	C16—H16	0.9500
C6—C9	1.504 (3)		
			
O2—S1—C1	107.96 (8)	C6—C9—H9B	109.5
O2—S1—C11	106.25 (9)	H9A—C9—H9B	109.5
C1—S1—C11	97.50 (8)	C6—C9—H9C	109.5
C8—O1—C7	106.70 (14)	H9A—C9—H9C	109.5
C8—C1—C2	107.61 (16)	H9B—C9—H9C	109.5
C8—C1—S1	123.11 (14)	C8—C10—H10A	109.5
C2—C1—S1	129.25 (14)	C8—C10—H10B	109.5
C3—C2—C7	119.70 (17)	H10A—C10—H10B	109.5
C3—C2—C1	135.32 (17)	C8—C10—H10C	109.5
C7—C2—C1	104.97 (16)	H10A—C10—H10C	109.5
C4—C3—C2	116.19 (17)	H10B—C10—H10C	109.5
C4—C3—H3	121.9	C16—C11—C12	120.99 (19)
C2—C3—H3	121.9	C16—C11—S1	118.35 (15)
C3—C4—C5	123.27 (18)	C12—C11—S1	120.50 (15)
C3—C4—Br1	117.87 (14)	C13—C12—C11	119.71 (19)
C5—C4—Br1	118.84 (14)	C13—C12—H12	120.1
C6—C5—C4	121.43 (18)	C11—C12—H12	120.1
C6—C5—H5	119.3	C14—C13—C12	118.06 (19)
C4—C5—H5	119.3	C14—C13—H13	121.0
C7—C6—C5	114.55 (17)	C12—C13—H13	121.0
C7—C6—C9	122.55 (18)	F1—C14—C15	118.2 (2)
C5—C6—C9	122.89 (18)	F1—C14—C13	118.4 (2)
O1—C7—C6	124.99 (16)	C15—C14—C13	123.4 (2)
O1—C7—C2	110.17 (15)	C14—C15—C16	118.3 (2)
C6—C7—C2	124.84 (17)	C14—C15—H15	120.8
C1—C8—O1	110.55 (16)	C16—C15—H15	120.8
C1—C8—C10	132.90 (17)	C11—C16—C15	119.53 (19)
O1—C8—C10	116.54 (16)	C11—C16—H16	120.2
C6—C9—H9A	109.5	C15—C16—H16	120.2
			
O2—S1—C1—C8	130.04 (16)	C1—C2—C7—O1	0.42 (19)
C11—S1—C1—C8	−120.12 (17)	C3—C2—C7—C6	−0.4 (3)
O2—S1—C1—C2	−47.76 (19)	C1—C2—C7—C6	−179.62 (17)
C11—S1—C1—C2	62.07 (18)	C2—C1—C8—O1	−0.9 (2)
C8—C1—C2—C3	−178.74 (19)	S1—C1—C8—O1	−179.16 (12)
S1—C1—C2—C3	−0.7 (3)	C2—C1—C8—C10	180.0 (2)
C8—C1—C2—C7	0.3 (2)	S1—C1—C8—C10	1.8 (3)
S1—C1—C2—C7	178.39 (14)	C7—O1—C8—C1	1.2 (2)
C7—C2—C3—C4	−0.8 (2)	C7—O1—C8—C10	−179.57 (16)
C1—C2—C3—C4	178.19 (19)	O2—S1—C11—C16	−17.77 (17)
C2—C3—C4—C5	1.0 (3)	C1—S1—C11—C16	−129.02 (16)
C2—C3—C4—Br1	179.66 (12)	O2—S1—C11—C12	166.73 (15)
C3—C4—C5—C6	−0.1 (3)	C1—S1—C11—C12	55.49 (16)
Br1—C4—C5—C6	−178.75 (13)	C16—C11—C12—C13	0.3 (3)
C4—C5—C6—C7	−1.0 (3)	S1—C11—C12—C13	175.68 (15)
C4—C5—C6—C9	178.10 (17)	C11—C12—C13—C14	−0.2 (3)
C8—O1—C7—C6	179.06 (17)	C12—C13—C14—F1	−179.34 (18)
C8—O1—C7—C2	−0.98 (19)	C12—C13—C14—C15	0.6 (3)
C5—C6—C7—O1	−178.80 (16)	F1—C14—C15—C16	178.93 (19)
C9—C6—C7—O1	2.1 (3)	C13—C14—C15—C16	−1.0 (3)
C5—C6—C7—C2	1.3 (3)	C12—C11—C16—C15	−0.7 (3)
C9—C6—C7—C2	−177.84 (17)	S1—C11—C16—C15	−176.19 (16)
C3—C2—C7—O1	179.65 (15)	C14—C15—C16—C11	1.0 (3)

### Hydrogen-bond geometry (Å, °)

**Table:**

*D*—H···*A*	*D*—H	H···*A*	*D*···*A*	*D*—H···*A*
C13—H13···O2^i^	0.95	2.41	3.259 (2)	148.

Symmetry codes: (i) *x*+1, *y*, *z*.

## References

[bb1] Akgul, Y. Y. & Anil, H. (2003). *Phytochemistry*, **63**, 939–943. 10.1016/s0031-9422(03)00357-1 12895543

[bb2] Aslam, S. N., Stevenson, P. C., Phythian, S. J., Veitch, N. C. & Hall, D. R. (2006). *Tetrahedron*, **62**, 4214–4226.

[bb3] Brandenburg, K. (1998). *DIAMOND* Crystal Impact GbR, Bonn, Germany.

[bb4] Bruker (2009). *APEX2* *SADABS* and *SAINT* Bruker AXS Inc., Madison, Wisconsin, USA.

[bb5] Choi, H. D., Seo, P. J., Son, B. W. & Lee, U. (2010*a*). *Acta Cryst.* E**66**, o1297. 10.1107/S1600536810016181 PMC2979595 21579394

[bb6] Choi, H. D., Seo, P. J., Son, B. W. & Lee, U. (2010*b*). *Acta Cryst.* E**66**, o1637. 10.1107/S1600536810021823 PMC3006853 21587866

[bb7] Choi, H. D., Seo, P. J., Son, B. W. & Lee, U. (2010*c*). *Acta Cryst.* E**66**, o1876. 10.1107/S1600536810024931 PMC3006988 21588071

[bb8] Farrugia, L. J. (1997). *J. Appl. Cryst.* **30**, 565.

[bb9] Galal, S. A., Abd El-All, A. S., Abdallah, M. M. & El-Diwani, H. I. (2009). *Bioorg. Med. Chem. Lett* **19**, 2420–2428. 10.1016/j.bmcl.2009.03.069 19345581

[bb10] Khan, M. W., Alam, M. J., Rashid, M. A. & Chowdhury, R. (2005). *Bioorg. Med. Chem* **13**, 4796–4805. 10.1016/j.bmc.2005.05.009 15964760

[bb11] Sheldrick, G. M. (2008). *Acta Cryst.* A**64**, 112–122. 10.1107/S0108767307043930 18156677

[bb12] Soekamto, N. H., Achmad, S. A., Ghisalberti, E. L., Hakim, E. H. & Syah, Y. M. (2003). *Phytochemistry*, **64**, 831–834. 10.1016/j.phytochem.2003.08.009 14559276

